# Supplementary material for: Simulating and Verifying a 2D/3D Laser Line Sensor Measurement Algorithm on CAD Models and Real Objects
Source: Sensors (Basel). 2024 Nov 20;24(22):7396. doi: 10.3390/s24227396 (PMC11598557; doi:10.3390/s24227396)
Supplement: Supplementary file 1 [file sensors-24-07396-s001.zip › Instructions_for_algorithm.pdf]

Generate 2D/3D laser line measurements on an imported CAD model: Instructions on how to use the algorithm.

1. Define the new coordinate system on the CAD model in modelling software such as SolidWorks. In this way you will easier define laser trajectory to successfully scan the CAD model.

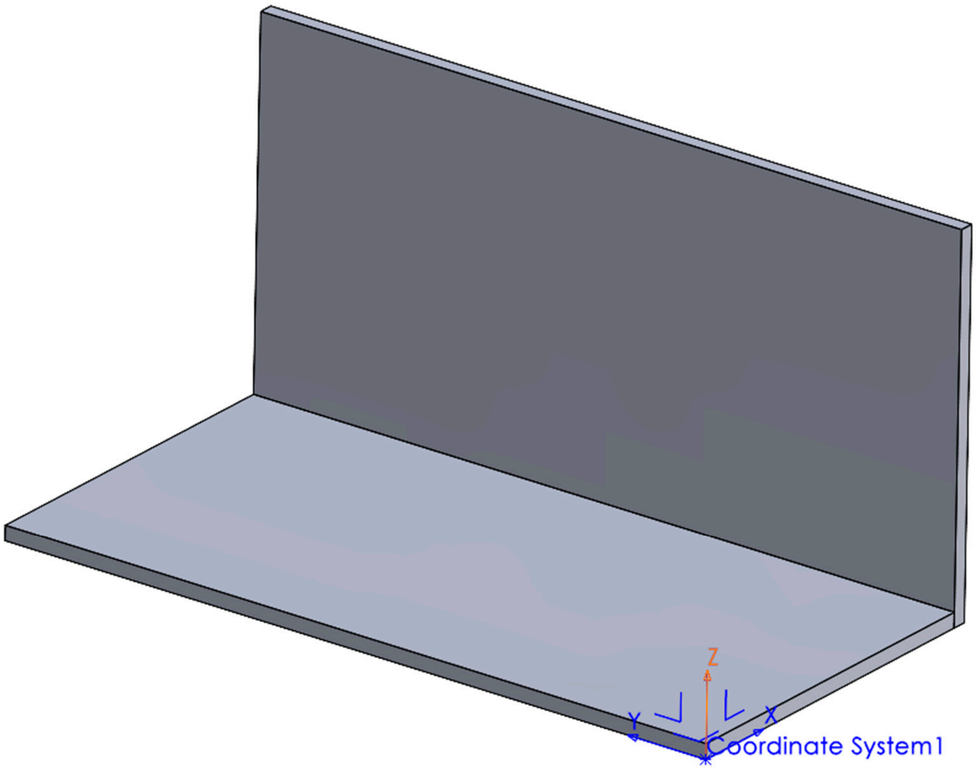

2. Save the CAD model in STL format where the new coordinate system must be selected. Use Fine resolution for STL model. This is the format that is supported by Matlab program.

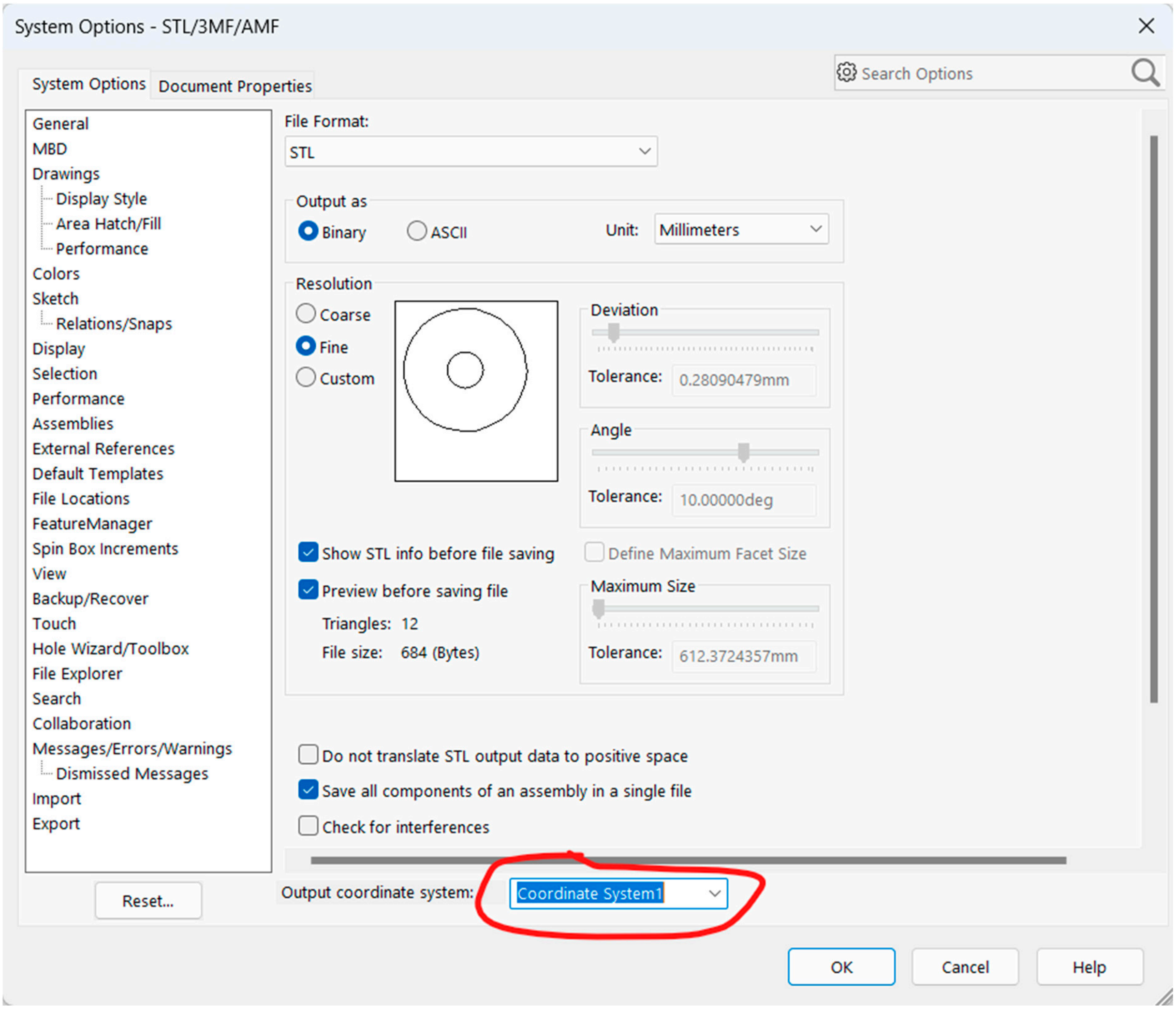

3. Import the STL CAD model into Matlab program. The STL CAD model should be in the working directory of the Matlab.

Define and import the CAD model

```
figure (10)

% Define stl file to be imported from the working directory
TR_CAD = stlread("L_250_250_500mm.STL");

% Plot 3D CAD model
trisurf(TR_CAD,FaceAlpha=0.5)
view([-52 25])
xlabel('X-axis [mm]')
ylabel('Y-axis [mm]')
zlabel('Z-axis [mm]')
axis equal
hold on
grid on
% Show global coodinate system
GCS_pos=[0 0 0]; % Global CS trans
GCS_ori = [0 0 0];% Global CS ori
q_GCS_ori_XYZ = eul2quat(GCS_ori,"XYZ");
GCS=plotTransforms(GCS_pos,q_GCS_ori_XYZ,'FrameSize',100);
```

4. Define 2D laser characteristics.

Define 2D laser characteristics

```
Work_rangeZ=[83 213];          % mm
Measur_rangeZ=130;              % mm
Measur_rangeX=[50 110];         % mm
% Laser resolution as per datasheet
Resul_Z=[3.2e-3 14e-3];       % mm
Resul_X=[26e-3 55e-3];          % mm
% Measurements per laser line
n=2048;
```

5. Define laser initial position and orientation in global coordinate system.

Define laser initial position and orientation in global coordinate system

```
L_x=100;
L_y=0;
L_z=150;
L_Rx=0*pi/180;
L_Ry=135*pi/180;
L_Rz=180*pi/180;
```

6. Define trajectory of the laser in global coordinate system
   m stands for number of scans
   Change other parameters if you want better resolution or change in rotation.

Define trajectory of the laser in global coordinate system

```
m=500;                            % Number of measurements per CAD model
L_trans_x=linspace(0,0,m);       % Translation of laser TCP in x
L_trans_y=linspace(0,m-1,m);          % Translation of laser TCP in y
L_trans_z=linspace(0,0,m);          % Translation of laser TCP in z
L_Rot_x=zeros(1,m);                 % Rotation of laser TCP around x
L_Rot_y=zeros(1,m);                 % Rotation of laser TCP around y
L_Rot_z=zeros(1,m);                 % Rotation of laser TCP around z
```

7. Define output interests

Define output interests

```
% Include resolution of the sensor? Yes (=1), No (=0)
Include_resolution=1;

% Plot laser working area? Yes (=1), No (=0) This increases
% time needed to perform the simulation
plotLaserWorkArea=1;

% XYZ measurements in Global CS? Yes (=1), No (=0)
XYZ_global=1;

% Generate point cloud in laser CS? Yes (=1), No (=0)
PointCloud_Laser_CS=1;

%Save point cloud data into PLY format? Yes (=1), No (=0)
PC_Save_Ply_L_CS=1;
% Define folder name:
PLY_fodler_name_L_CS='PC_L_L_250_250_500mm_01';

% Generate point cloud in global CS? Yes (=1), No (=0)
PointCloud_Global_CS=1;
%Save point cloud data into PLY format? Yes (=1), No (=0)
PC_Save_Ply_G_CS=1;
% Define folder name:
PLY_fodler_name_G_CS='PC_G_L_250_250_500mm_01';

% Plot results as 2D image histogram? Yes (=1), No (=0)
TwoD_histogram_plot=0;

% Save results in Excel file? Yes (=1), No (=0)
Save_res_in_Excel=1;
% Define Excel file
filename = 'Sim_PC_L_250_250_500mm_01.xlsx';
```

8. The rest of the code is to perform simulated measurements.
